# Phenotypic and genotypic characteristics of *Pseudomonas aeruginosa* isolated from cystic fibrosis patients with chronic infections

**DOI:** 10.1038/s41598-023-39005-9

**Published:** 2023-07-20

**Authors:** Agnieszka Iwańska, Elżbieta Anna Trafny, Michał Czopowicz, Ewa Augustynowicz-Kopeć

**Affiliations:** 1 Department of Microbiology, National Tuberculosis and Lung Diseases Research Institute, Warsaw, Poland; 2 Biomedical Engineering Centre, Institute of Optoelectronics, Military University of Technology, Warsaw, Poland; 3 Division of Veterinary Epidemiology and Economics, Institute of Veterinary Medicine, Warsaw University of Life Sciences-SGGW, Warsaw, Poland

**Keywords:** Microbiology, Clinical microbiology

## Abstract

Patients with cystic fibrosis are predisposed to chronic respiratory tract infections caused by *Pseudomonas aeruginosa*. As the disease progresses, the microorganism diversifies into genotypically and phenotypically different strains which may coexist in the patient's airways for years. Adaptation of the microorganism to the airways of patients with cystic fibrosis probably occurs in response to the host's airway environment, the elements of the immune system and antibiotic therapy. Due to the chronic persistence of the microorganism in the airways, a comprehensive molecular analysis was conducted. The analysis included 120 strains isolated from 10 adult cystic fibrosis patients with chronic *P. aeruginosa* infection. The aim of the study was to analyze the molecular patterns of *P. aeruginosa* strains and to trace their transmission in the population of cystic fibrosis patients, as well as to study a relationship of the disease with specific phenotypic features. In the research, a genotypic analysis of *P. aeruginosa* was performed using pulsed-field gel electrophoresis. The results of a number of phenotypic features of the strains were added to the outcomes of the molecular studies. As a result, 28 different genotypes were distinguished. The study also showed cross-transmission of strains between patients. 3 transmissible clusters were identified, including IG1 and IG2 clusters with 9 strains of *P. aeruginosa* each, obtained from 2 patients and IG3 cluster with 6 strains of *P. aeruginosa* isolated from 3 patients. Moreover, it was found that in some patients, several unrelated strains of *P. aeruginosa* may transiently or permanently infect the respiratory tract. A comprehensive understanding of the *P. aeruginosa* adaptation may help to develop more effective antimicrobial therapies and to identify new targets for future drugs in order to prevent progression of the infection to chronic stages.

## Introduction

Cystic fibrosis (CF) is the most common genetic disease in the Caucasian population, inherited in an autosomal recessive manner. Data from the European CF Patient Registry shows that over 52,000 people are affected in Europe^1^.

The disease is caused by a mutation in the CFTR (cystic fibrosis transmembrane conductance regulator) gene, located on the long arm of chromosome 7. The mutation results in the abnormal production of the CFTR protein and, consequently, a disorder of ion transport in tissues of the epithelial origin, mainly in the respiratory system, digestive system, reproductive system and secretory glands^2–5^. Currently, more than 2100 mutations affect the CFTR gene, of which 400 lead to the development of the disease^6^. Over the last decade, the median survival of patients has significantly increased, which is considerably influenced by better understanding of the mechanisms of the disease. Despite improvements in the treatment and patient care, bacterial respiratory infections continue to be a significant clinical problem in CF.

Chronic and recurrent acute respiratory infections play a key role in the development of chronic bronchopulmonary disease with progressive tissue damage and impaired lung function^1, 5, 7–9^.

*Pseudomonas aeruginosa* is the dominant microorganism in cystic fibrosis patients with infections. It is estimated that chronic infection develops in 50–70% of patients before the age of 30 years^7, 10, 11^.

During childhood, patients with cystic fibrosis usually experience incidental episodes of infection; eradication is usually effective at this stage^5, 10, 11^. However, as the disease progresses, CF patients experience more reinfections. Molecular studies demonstrated that each subsequent infection may be caused by either a strain with the genotype of the first infection or a new strain acquired from the environment^12^.

The prevailing view is that chronic *P. aeruginosa* infection is caused by a dominant clone which adapts to the conditions in the airways and evolves through microadaptation into multiple sublines of the original clone^13–15^. Co-infection with two or more different *P. aeruginosa* genotypes has also been reported^16, 17^. The source of infections is usually in the hospital or home environment, through nebulizers or cross-infections between patients^4, 10, 18, 19^.

*Pseudomonas aeruginosa* has a complex set of virulence factors responsible for the pathogenesis of infections. These factors and their regulatory pathways are the subject of intense research. Researchers around the world are making efforts to better understand the virulence and adaptation mechanisms of *P. aeruginosa* so that they can be used as potential therapeutic targets^20–22^.

The aim of the study was to determine the genotypic variation of 120 strains of *P. aeruginosa* obtained from 10 CF patients with chronic infections and to assess the possible transmission of *P. aeruginosa* between patients, as well as to study the relationship of the disease with specific phenotypic features.

## Results

### Patients

The strains of *P. aeruginosa* came from 6 women and 4 men. The age of patients at the time of study enrolment ranged from 18 to 34 years, with the median of 21.5 years (women—20.5 years, men—23.5 years). As for the F508del mutation, 4 patients were homozygous and 4 heterozygous; in the case of 2 patients, no information was obtained. The mean forced expiratory volume in 1 s value at the time of study enrolment was 43.9, and the range was between 24.1 and 73.6 of the normal value (Table 1).

**Table 1:** Clinical characteristics of the ten patients treated at the Institute of Tuberculosis and Lung Diseases between 2012 and 2019 (N = 10).

Patient no.	Patient	Sex	Age at start of study [years]	CFTR classification	FEV_1_% predicted, mean	Number of hospitalizations	Number of outpatient visits	Number of exacerbations	Number of *P. aeruginosa* isolates^a^
1	P1	W	21	F508del/F508del	73.6	56	11	11	13
2	P2	W	22	F508del/3849+10 kb C>T	69.8	80	25	24	17
3	P3	W	18	F508del/R553X	30.4	6	31	32	9
4	P4	W	20	F508del/1717-1	54.8	39	25	24	10
5	P5	M	24	No data	24.1	47	31	30	13
6	P6	M	23	F508del/F508del	36.6	13	20	19	14
7	P7	M	34	F508del/F508del	26.5	43	4	4	14
8	P8	M	19	F508del/3849+10	40.1	61	16	16	8
9	P9	W	18	No data	52.2	23	24	24	8
10	P10	W	22	F508del/F508del	30.8	46	13	14	14

*CFTR* cystic fibrosis transmembrane conductance regulator, *FEV*_*1*_ forced expiratory volume in 1 s.

^a^Isolate, single colony obtained from the selective agar plates of plated sputum samples from the patient in the following periods.

### Genetic relationship between *P. aeruginosa* isolates

**Figure 1:**
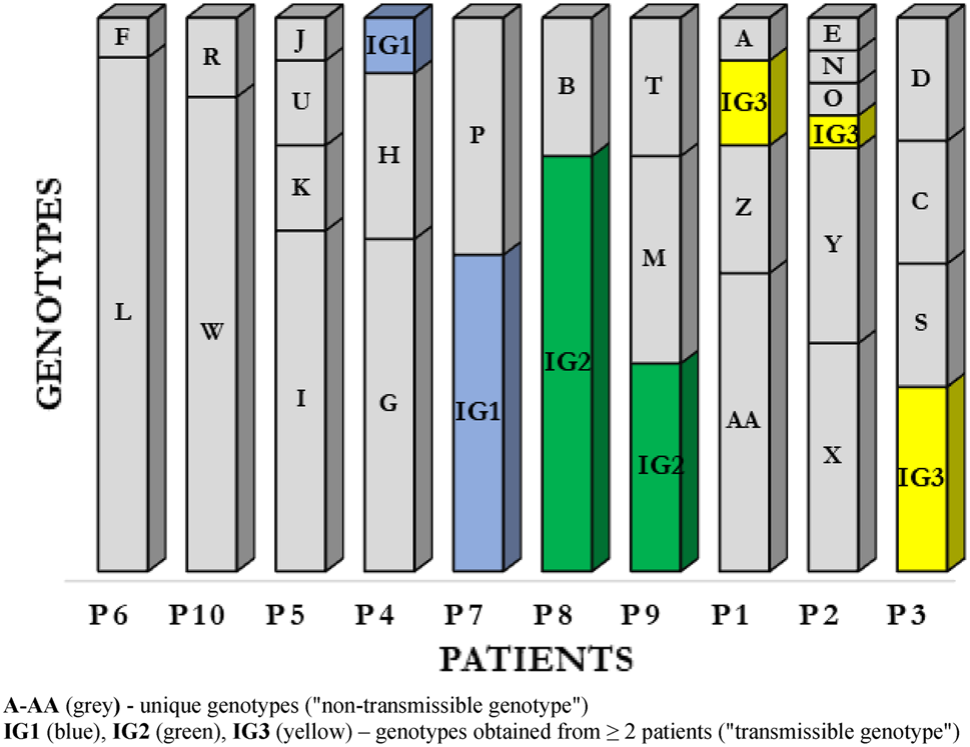
Distribution of 120 *P. aeruginosa* isolates belonging to different PFGE types among 10 cystic fibrosis patients.

Pulsed-field gel electrophoresis (PFGE) was performed for all 120 *P. aeruginosa* isolates (Supplemental Material; Fig. 1). By setting the similarity threshold at 90%, 28 different PFGE profiles were identified, including 25 (89.3%) genotypes classified as unique ("non-transmissible") strains isolated from single patients (marked consecutively from A to AA); the remaining 3 (10.7%) profiles were determined as common ("transmissible") genotypes obtained from at least 2 patients (IG1, IG2, IG3) (Fig. 1).

### Differentiation of *P. aeruginosa* genotypes in adult cystic fibrosis patients with chronic infections

Based on the number of genotypes during the study period, the patients were divided into 2 groups: (1) with "genotypic stability of strains" (maintenance of the same genotype or 2 genotypes; has also been included patient P4 with 2 predominant genotypes and with 1 genotype, which occurred only once) and (2) with "genotypic variation of strains" (different genotypes during the study period). 5 patients were assigned to both groups.

Our data show that 3 CF patients (P6, P8 and P10) were colonized with unique clones during the study period: L, IG2 and W respectively. In this patients observed only transient colonization with other genotypes (incidental genotypes). In patient P7 we observed coexistence two *P. aeruginosa* genotypes: IG1 and P. From patient P4, we observed a complete replacement of the H genotype by the G genotype and obtained IG1 genotype, identical to that found in patient P7.

In the group of patients with chronic infections and genotypic variation, we observed overall high level of *P. aeruginosa* genotype diversity. The *P. aeruginosa* population dynamics in patient P2 was of special interest, with 6 different genotypes. Patients P1, P3 and P5 showed 4 genotypes each, and patient P9 had 3 types with different restriction patterns.

Results show co-existence of several genotypes was observed, which may indicate the presence of strains with different molecular patterns at the same time (Fig. 1).

### Comparative analysis of patients with stability and genotypic variation during chronic infection

Comparing data on gender, age, CFTR gene mutation and comorbidities, no significant differences were found between both groups of patients. However, in the group with a genotypic variation of strains, a greater number of cycles of intravenous antibiotic therapy (26 vs. 16 cycles) and a tendency to more frequent and longer hospitalizations were observed; these differences were not statistically significant (Table 2).

**Table 2:** Comparative analysis of patients with stability and genotypic variation during chronic infection (N = 10).

Clinical information		Stability genotypic	Variation genotypic	p-value
Number of patients		5	5	–
Age at diagnosis in years^b^		22 (19–34)	21 (18–24)	0.421
Male gender^a^		3 (60.0)	1 (20.0)	0.524
CFTR classification^c^	ΔF508 homozygous (%)	3 (60.0)	1 (20.0)	–
	ΔF508 heterozygous (%)	2 (40.0)	2 (40.0)	–
	no data	–	2 (40.0)	–
FEV_1_ predicted %^b^		36.6 (26.5–54.8)	52.2 (24.1–73.6)	0.690
BMI (kg/m^2^)^b^		18.2 (16.9–19.6)	18.8 (14.4–21.6)	0.841
Cystic fibrosis-related diabetes (%)^a,d^		4 (80.0)	2 (40.0)	0.524
Pancreatic insufficiency^a,e^		4 (80.0)	4 (80.0)	0.999
Number of hospitalizations during the study period^b^		16 (4–25)	25 (11–31)	0.151
Total number of days hospitalization^b^		240 (59–336)	489 (151–750)	0.095
Number of outpatient visits during the study period^b^		43 (13–61)	47 (6–80)	0.841
Number of cycles of intravenous therapy^b^		16 (4–24)	26 (11–32)	0.095

*CFTR* cystic fibrosis transmembrane conductance regulator, *BMI* body mass index, *FEV*_*1*_ forced expiratory volume in 1 s.

^a^n (%) and the Fisher exact test; ^b^median (range) and the Mann–Whitney *U* test; ^c^homozygous: both alleles containing the delta F508 mutation; Heterozygous: one allele containing the delta F508 mutation; ^d^cystic fibrosis-related diabetes: use of insulin; ^e^pancreatic insufficiency: use of pancreatic enzymes.

### Transmissible clusters

Transmissible clusters represented by the profiles IG1, IG2 and IG3 of *P. aeruginosa* were isolated from 7 patients. The IG1 cluster contained 9 *P. aeruginosa* strains from 2 patients, including 8 strains from just 1 patient P7. Similarly, the IG2 cluster contained 9 *P. aeruginosa* strains from 2 patients, including 6 strains isolated from 1 patient P8. The IG3 cluster contained *P. aeruginosa* isolated from 3 patients—P3, P1 and P2, from whom 3, 2 and 1 strain(s) were obtained, respectively.

The epidemiological investigation revealed that hospitalization played an important role in transmission of the strains (Table 3).

**Table 3:** Periods of hospitalization of patients with transmission clusters.

Cluster	Patient	Isolates	Date of isolation	Hospitalization in the same period
IG1	P7	P7-1	11-2012	31.05.2019–03.06.2019
		P7-2	02-2012	
		P7-3	04-2014	
		P7-6	02-2017	
		P7-7	02-2017	
		P7-8	08-2017	
		P7-9	07-2018	
		P7-10	07-2018	
	P4	P4-7	06-2019	
IG2	P8	P8-1	11-2013	04.12.2013–06.12.2013
		P8-2	11-2014	
		P8-3	01-2015	
		P8-4	05-2016	
		P8-5	10-2017	
		P8-7	07-2019	
	P9	P9-1	12-2013	
		P9-2	02-2014	
		P9-3	08-2014	
IG3	P2	P2-6	10-2017	17.04.2018–23.04.2018 In addition P3 and P2 were hospitalized together by three times (07/2018; 09/2018; 11/2018)
	P1	P1-9	11-2018	
		P1-10	02-2019	
	P3	P3-7	02-2019	
		P3-8	08-2019	
		P3-9	09-2019	

The source of strains with an identical genotype could be joint hospitalization, i.e. contact between patients. However, due to the lack of environmental studies, we cannot rule out the environmental source of the transmission of *P.* *aeruginosa* strains.

### Comparison of virulence factors in transmissible and non-transmissible *P. aeruginosa* strains

The expression of phenotypic features was analyzed between *P. aeruginosa* strains belonging to common and unique clones.

Transmissible strains, with IG1, IG2, IG3 profiles, were combined into one group due to the small sample size of individual clones.

- Genotypes transmitted between patients (IG1, IG2, IG3)—24 transmissible strains.
- Unique genotypes—96 non-transmissible strains.

**Figure 2:**
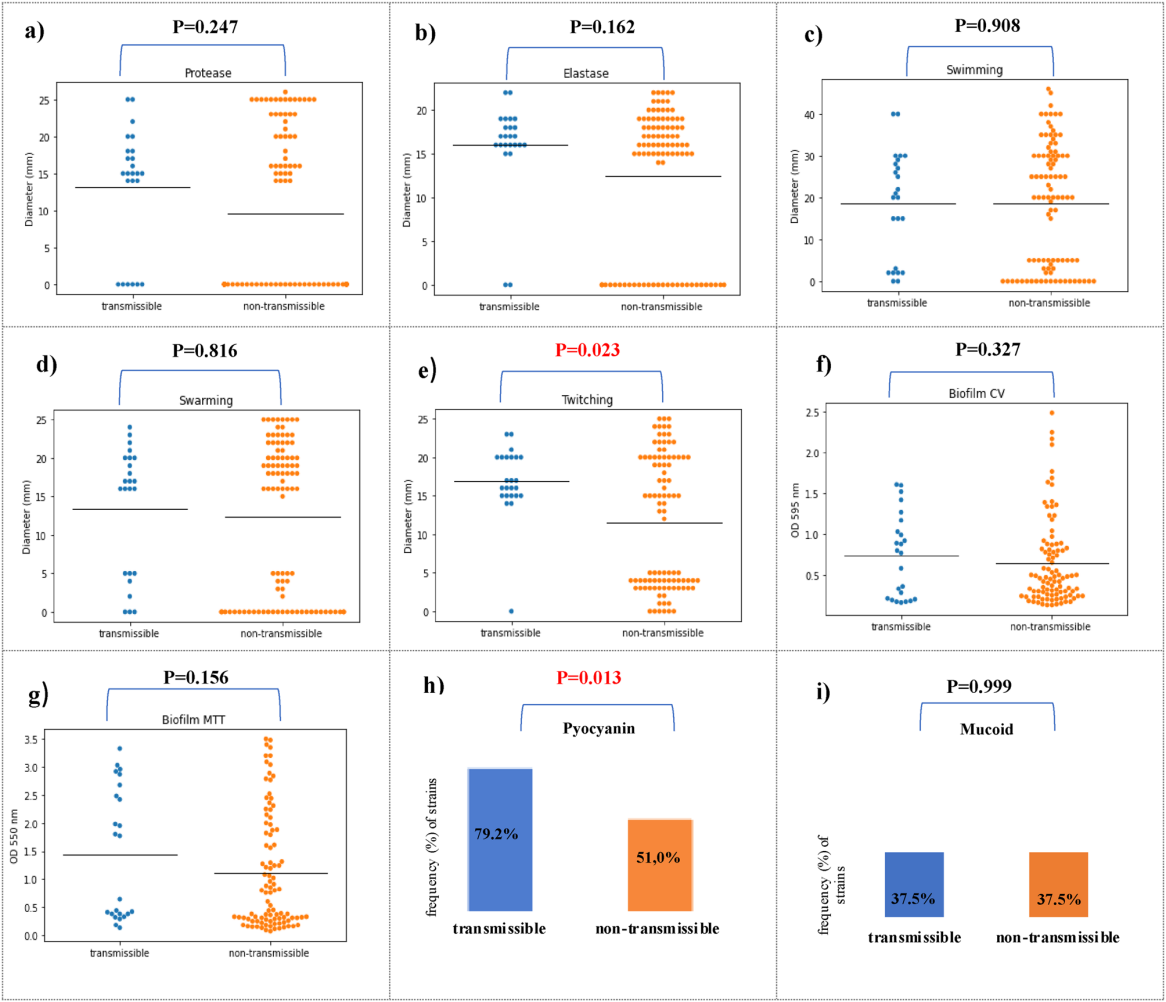
Comparison of *P. aeruginosa* isolates between transmissible (n = 24) (blue) and non-transmissible (n = 96) (orange) *P. aeruginosa* isolates. Isolates were characterized for protease (**a**), elastase (**b**), swimming (**c**), swarming (**d**) and twitching (**e**) motility; biofilm CV (biomass) (**f**); MTT cell viability assay (**g**), pyocyanin production (**h**) and mucoid phenotype (**i**). Black horizontal bars represent the mean of each group.

First, we compared the frequency distribution of virulence factors between transmissible and non-transsmisible isolates. The frequency analysis of virulence factors showed that the percentage of strains capable of producing protease (75.0% vs 46.9%, p = 0,014), elastase (91.7% vs 70.8%, p = 0,035), produce pyocyanin (79.2% vs. 51.0%, p = 0.013) and twitching motility (95.8% vs 52.1%, p < 0.001) was significantly higher compared to non-transmissible strains (Table 4, Fig. 2).

**Table 4:** Comparison frequency of virulence factors between transmissible and non-transmissible strains.

Virulence factor	Transmissible (N = 24)	Non-transmissible (N = 96)	p-value
Mucoid phenotype	9 (37.5)	36 (37.5)	0.999
Biofilm CV	14 (58.3)	55 (57.3)	0.926
Biofilm MTT	19 (79.2)	59 (61.5)	0.104
Protease	18 (75.0) ↑	45 (46.9)	0.014
Elastase	22 (91.7) ↑	68 (70.8)	0.035
Swimming	17 (70.8)	59 (61.5)	0.394
Swarming	16 (66.7)	56 (58.3)	0.456
Twitching	23 (95.8) ↑	50 (52.1)	< 0.001
Pyocyanin	19 (79.2) ↑	49 (51.0)	0.013

The research into the activity of virulence factors revealed the varied range of expression for each phenotypic trait in both groups. Analysing the activity of key virulence factors between transmissible and non-transmissible strains showed that twitching motility (16.9 mm vs. 11.5 mm, p = 0.023) was statistically significantly higher for transmissible strains. The study showed that the transmission strains in comparison to the non-transmission had a higher average activity of protease (13.1 mm vs. 9.6 mm, p = 0.247) and elastase (16.0 mm vs. 12.5 mm, p = 0.162), however, it was not a statistical trend. The strains from both groups showed a similar ability to swim (18.5 mm vs. 18.5 mm, p = 0.908), to swarm (13.5 mm vs.12.3 mm, p = 0.816) and to form biofilm in the tests using crystal violet (0.74 vs. 0.6, p = 0.327) and MTT (1.5 vs. 1.1, p = 0.156) (Fig. 2**)**.

The analysis of the drug resistance phenotype showed no significant differences between the two groups of strains. In both groups, resistance to amikacin was most frequently observed—in 18 transmissible strains (75%) and in 72 non-transmissible strains (75%); resistance to ciprofloxacin—in 15 transmissible strains (62.5%) and in 61 non-transmissible strains (63.5%). Variations in resistance to colistin have been found. Common genotypes did not show resistance to this drug. In the group of non-transmissible genotypes, 7.3% (7) of strains were resistant to colistin (Table 5). This data indicates the lack of the specific resistance pattern of *P. aeruginosa* isolates among transmissible clusters.

**Table 5:** Comparison between antimicrobial resistance of transmissible and non-transmissible genotypes.

*P. aeruginosa* strains	Resistance, n (%)						
	TZP	CAZ	MEM	AK	TOB	CIP	CL
Transmissible (n = 24)	8 (33.3)	11 (45.8)	11 (45.8)	18 (75.0)	13 (54.2)	15 (62.5)	0 (0)
Non-transmissible (n = 96)	34 (35.4)	53 (55.2)	51 (53.1)	72 (75.0)	56 (58.3)	61 (63.5)	7 (7.3)

*n* number of strains, *TZP* piperacillin*/*tazobactam, *CAZ* ceftazidime, *MEM* meropenem, *AK* amikacin, *TOB* tobramycin, *CIP* ciprofloxacin, *CL* colistin.

Statistical analysis indicated that transmission strains of *P. aeruginosa* could be differentiated from non-transmission strains based on their higher ability to twitching motility and produce pyocyanin.

## Discussion

In this study, the analysis included 120 *P. aeruginosa* strains obtained from 10 adult CF patients treated at the Institute of Tuberculosis and Lung Diseases in Warsaw.

Among the 120 strains, 28 genotypes were distinguished, which indicates significant genotypic variation of the *P. aeruginosa* strains isolated from patients with cystic fibrosis. Most of the patients were infected with the organisms having unique, non-transmissible restriction patterns (96 strains). Three clusters with the same molecular patterns were identified (24 strains). The IG1 and IG2 clusters consisted of 9 strains each isolated from 2 patients, and the IG3 cluster comprised 6 strains from 3 patients, which suggests the possible cross-infection in the population of patients with CF. All patients had documented joint stays in the hospital wards of the Institute of Tuberculosis and Lung Diseases, where the transmission was most likely. Because no environmental studies were carried out in this period, a reservoir of transmissible strains located in the hospital environment cannot be ruled out.

The analysis of molecular diversity of 1352 *P. aeruginosa* strains isolated from 338 CF patients treated in an Italian center identified 43 common clusters and 214 profiles unique to individual patients. Common clusters included *P. aeruginosa* strains isolated from 2 (0.6%) to 12 (3.6%) patients; their presence indicated possible transmission of *P. aeruginosa* between patients in the clinic. However, the authors of the study assessed the risk of *P. aeruginosa* cross-infection in the study population as extremely low, emphasizing the importance of infection control procedures and *P. aeruginosa* first eradication strategies to reduce transmission rates^23^.

Similarly, the assessment of *P. aeruginosa* diversity and prevalence in patients treated at the Gothenburg cystic fibrosis center over a 10-year follow-up period showed a wide variety of strains, with 72% of the patients having unique strains of *P. aeruginosa*. The authors of the study concluded that the patients were most likely infected with strains from the environment^24^.

Our results confirm that the majority of patients were infected with non-transmissible *P. aeruginosa* strains. However, the presence of 3 transmissible clusters indicates a necessity for monitoring transmissible genotypes due to their possible spread in the population of patients with cystic fibrosis. The use of infection control procedures, such as triage and hygiene standards, is necessary in clinical practice. Infection control recommendations for patients with cystic fibrosis apply to hospital, outpatient and home conditions^7^.

Numerous studies have shown that intermittent infections, during which *P. aeruginosa* is eradicated, are followed by chronic infection with a dominant clone which adapts to the conditions in the airways of patients, and changes in this genotype are very rare^13–15^. Our study results challenge the idea that patients with chronic cystic fibrosis are infected with a single clonal lineage of *P. aeruginosa*.

Through our analysis of the *P. aeruginosa* population and the dynamics of chronic infections in 10 patients with CF treated at the Institute of Tuberculosis and Lung Diseases showed that during the study period, at least 2 genotypically different strains of *P. aeruginosa* were observed in patients with CF. Based on the relative number of genotypes identified during the study period, the patients were divided into 2 groups—genotypically stable (5 patients) and diverse (5 patients) *P. aeruginosa*. In the group of patients with genotypic stability, 3 patients had permanent *P. aeruginosa* genotype with an occasional transient genotype, 1 patient had co-infection with two genotypes, and 1 patient showed clonal replacement. In the group of patients with genotypic variation of strains, the heterogeneity of the *P. aeruginosa* population was observed, with the coexistence of several genotypic lines. Moreover, in 5 patients, in 7 cases, *P. aeruginosa* strains with different genotypes were obtained from the same sputum sample, suggesting that genotypically different *P. aeruginosa* are not mutually exclusive. Our results prove the co-occurrence of genotypically different *P. aeruginosa* strains in the airways of patients with CF.

In the study conducted by Jelsbak et al., the dynamics of *P. aeruginosa* infections in CF patients was analyzed, from early infections in children to chronic infections in adults. The authors showed the high level of *P. aeruginosa* genotypic variation in the early stages of the disease. This indicates that the acquisition of unique *P. aeruginosa* strains occurs independently, presumably from different environmental reservoirs. However, in the group of patients with chronic *P. aeruginosa* infection lasting over 12 years, among 45 strains isolated from 7 patients, 5 genotypes were found, with significant overrepresentation of 2 clones. The authors suggested that the dominant clones retained the ability to transmit between CF patients due to their strong selectivity, which allowed them to infect the patients' lungs^25^.

In a 9-year follow-up period, Kalferstova et al. demonstrated that in a population of 131 patients, 110 (84.0%) were infected with the strain of the same genotype, while only 21 patients (16.0%) developed a new strain^26^. Another study found that, 20 years after the first isolation, more than half of the population of patients were infected with the same *P.* *aeruginosa* clone^14^.

Similar results to those presented in this paper were obtained by scientists from Canada who assessed the diversity of the sequence types (ST) of 1537 *P. aeruginosa* strains in 402 pediatric and adult CF patients. The authors observed that 43% of the infections were stable, and 57% showed a variety of sequence types in the study period. In the diverse group, 28% were dynamic and 29% reversible, i.e. after ST change, there was a return to previously isolated sequence types^27^.

The long-term 8-year analysis conducted in our research allowed us to study the changing genotypic patterns of *P. aeruginosa* in patients. The study was retrospective. Its aim was to conduct observational research using the strains collected in the strain bank of the Department of Microbiology of the Institute of Tuberculosis and Lung Diseases. Due to the current procedure, dominant morphotypes isolated from clinical materials, obtained from the respiratory tract of patients with cystic fibrosis, were banked. Despite the above limitations, genotypic variation could be higher because not all genotypes could be detected, and this indicates the need to extend microbiological diagnostics.

Although sputum testing appears to be a reliable means of detecting *P. aeruginosa* in the lower respiratory tract, in CF patients, different areas of the lungs may be colonized by various isolates. Various strains may be predominant in different regions of the lungs and are subject to changing environmental conditions, such as oxygen deprivation. These limitations may lead to underestimation of dominant genotypes and the genetic variation of *P. aeruginosa* strains in CF patients.

Determining population structure (stability or genotypic variation) is probably influenced not only by the mechanism of *P. aeruginosa* genotypic competition but also by host-related factors, such as antibiotic therapy, immune system function and other stressors^25^. Aggressive antibiotic therapy in patients with CF can temporarily eradicate *P. aeruginosa*, but this is followed by new episodes of infection. The data indicates that reinfection may be caused by strains with a different restriction pattern, by the same clone that may have been transiently undetected in sputum culture, or by reinfection from an environmental source. There are also reports suggesting that the paranasal sinuses are the source of *P. aeruginosa* strains^28, 29^.

Our study also evaluated the correlation between stability, genotypic variation and the clinical characteristics of patients. Among patients with a dynamic genotypic variation of strains, higher frequency and duration of hospitalization, as well as more cycles of intravenous therapy, were observed; however, these differences were not statistically significant. Therefore, the question arises whether the genotypic variation of *P. aeruginosa* has an impact on deterioration of the clinical condition of patients or, alternatively, whether it results from greater exposure to *P. aeruginosa* strains in the hospital environment.

Virulence factors may affect not only the ability of *P. aeruginosa* to persist in the host organism but also to increase the ability of the microorganism to spread among patients with CF^15^. Therefore, the authors decided to check whether there are any virulence factors of easier transmission of strains. Twenty four transmissible and 96 non-transmissible strains were analyzed for a distinctive phenotypic marker. Due to the small sample size in individual clusters, the transmissible group included all strains belonging to the 3 clusters (IG1, IG2 and IG3).

Analysing the activity of key virulence factors between transmissible and non-transmissible strains showed that twitching motility (p = 0.023) and pyocyanin production (p < 0.001) were statistically significantly higher for transmissible strains.

Pyocyanin, a phenazine pigment produced by *P. aeruginosa*, has long been important virulence factor in the pathogenesis of *P. aeruginosa* infections. Our study shows that pyocyanin production may be assosiated with transmission of strains of *P. aeruginosa* among CF patients. It was demonstrated that transmission strains produced the pyocyanin significantly more frequently compared to non-transmission strains (79.2% vs 51.0%, p = 0.013).

It is consistent with work done by Fothergill et al., which showed that many isolates exhibit over-production of pyocyanin, which has a number of toxic effects directly relevant to cystic fibrosis^15^.

The higher activity of *P. aeruginosa* capable of twitching (p = 0.023) among transmissible strains, as compared to non-transmissible strains, as found in our study, may indicate the involvement of type IV pili in the transmission of strains between patients. This seems understandable because type IV pili participate in the development of infection by adhering to host cells in the initial phase and are one of the most necessary elements of *P. aeruginosa* infection^30^.

*Pseudomonas aeruginosa* produces a number of proteases directly damage host tissue and subvert immune cell functions. The most widely studied are alkaline protease or elastase. The study of proteases is important to better understand of *P. aeruginosa* infection in CF patient^30^. Our study showed that, compared to non-transmissible strains, transmissible strains are characterized by a higher average activity of protease (13.1 mm vs. 9.6 mm, p = 0.247) and elastase (16.0 mm vs. 12.5 mm, p = 0.162); however, this was not a statistically significant trend. Therefore, it seems possible that transmissible strains have developed common mechanisms in which virulence factors, such as alkaline protease and elastase, are not subjected to expression modification, which favors the spread between patients.

Our results support evidence from other studies showing that protease and elastase activity may mediate increased transmission of strains among CF patients. Similar results were obtained in in vitro studies conducted using the Australian epidemic strains AES-1 and AES-2, which showed the increased production of virulence factors, such as the activity of protease and elastase, compared to non-epidemic strains isolated from CF patients, although the differences were not statistically significant^31^.

In their study, Duong et al. compared the activity of virulence factors of epidemic and non-epidemic strains and showed that local strains had a higher average activity of elastase and biofilm biomass^32^.

The analysis revealed that, contrary to non-transmissible strains, transmissible strains retain virulence factors—markers of acute infection, such as protease and elastase. These findings are consistent with the recent reports of an epidemic LES strain that has been shown to have increased elastase and pyocyanin activity^33^.

In conclusion, our study suggest that was no widespread outbreak at the National Institute of Tuberculosis and Lung Disease, and the majority of the genetic diversity in *P. aeruginosa* was observed between patients. We observed cross-transmission in a few of the cases. In light of the potential cross-infection associated with the acquisition of common *P. aeruginosa* strains, analysis of virulence factors in transmissible strains is crucial. Advances in the understanding of the increased transmission of *P. aeruginosa* strains are critical to the development of new therapeutics to treat infected CF patients and strategies to prevent transmission.

This is the first such analysis of strains obtained from adult patients with CF in Poland. Therefore, we believe that our study can serve as a reference point for future research and can help to gain a more complete understanding of *P. aeruginosa* infections in CF patients.

## Methods

### Clinical isolates

The subject of the study was a collection of 120 *P. aeruginosa* strains isolated from 96 clinical materials obtained from 10 adults (≥ 18 years of age) suffering from CF, from January 2012 to December 2019. Table 1 presents the characteristics of the subjects.

The retrospective and prospective analysis of *P. aeruginosa* strains was approved by the Bioethics Committee of the Institute of Tuberculosis and Lung Diseases in Warsaw (No. KB-74/2019).

The materials were processed in accordance with the principles of applicable laboratory procedures, inoculated directly on agar media: Columbia supplemented with 5% sheep blood, MacConkey and Cetrimide (bioMérieux, Marcy-l'Étoile France). The cultures were incubated for 24 to 48 h at 37 °C. Preliminary identification of *P. aeruginosa* was performed by routine microbiological methods (oxidase, pigment production, growth at 42 °C) and confirmed with the commercial VITEK®2Compact system (bioMérieux). *P. aeruginosa* strains (dominant morphotypes) were stored at − 80 °C in the MicrobankTM system (Pro-Lab) or in 50% (v/v) glycerol (Stanlab, Poland) in the strain bank of the Department of Microbiology at the Institute of Tuberculosis and Lung Diseases in Warsaw.

### Isolation of genomic DNA from bacterial cultures and pulsed-field gel electrophoresis (PFGE)

A suspension (density 4 of the McFarland standard) was prepared from a culture of *P. aeruginosa* on Columbia agar. 1 ml of the suspension was then centrifuged for 5 min at 12,000 rpm. The sediment was resuspended in 150 µl of cell suspension buffer (1 M Tris, 0,5 M EDTA, pH 8,0) and incubated for 10 min at 55 °C, followed by the addition of 20 µl of proteinase K (20 mg/ml; A&A Biotechnology). The suspension was combined with 1.5% agarose (SeaKem^®^Gold, LonzaTM) and filled into the wells (Bio-Rad, USA). Solidified agarose blocks were transferred to tubes with a 2.5 ml lysis buffer (1 M Tris, 0.5 M EDTA pH 8,0, 1% Sarcosyl) 12 µl proteinase K and 3 µl RNAse (10 mg/µl; A&A Biotechnology). After 2-h lysis at 55 °C, the buffer was removed, and the blocks were washed once in dH_2_O and three times in a TE buffer (10 mM Tris, 1 mM EDTA, pH 8,0). The DNA block was transferred to a tube with a 50 µl *Spe*I-specific buffer (20,000 U/ml; New England BioLabs, USA) and incubated for 30 min at room temperature. The block was then placed in 49 µl of *Spe*I—specific buffer and 1 µl of *Spe*I enzyme (New England BioLabs, USA) and incubated at 37 °C for 3 h. Pulsed-field gel electrophoresis (PFGE) of *P. aeruginosa* strains was performed using the CHEF MAPPER™ apparatus (Bio-Rad, USA), in the modification of the protocol of Hu et al.^34^. Electrophoretic separation was performed in 1% pulse field certified agarose (Bio-Rad). Digested blocks were placed in gel wells and flooded with 1% agarose. Lambda Ladder PFG Marker with a range of 48.5–1.018 kb (New England BioLabs, USA) was used as a size standard.

Patterns of DNA bands were analyzed with Bionumerics Software (bioMérieux, Marcy-l'Étoile France) using the Dice similarity coefficient and the UPGMA clustering technique (position tolerance and optimization 1.5%). Individual types were defined according to the interpretative criteria proposed by Tenover et al.^35^. A cluster was defined as a group of isolates with ≥ 90% PFGE profile similarity (Supplemental Material, Fig. 1).

### Phenotypic tests

Bacteria were cultured in tryptic soy broth (TSB; Difco, Sparks, MD) for 16–18 h in a shaking incubator at 37 °C. All tests were performed as 3 independent trials, with 3 replicates per trial, resulting in 9 measurements per trait per isolate. *P. aeruginosa* ATCC 27853, *Escherichia coli* NCTC 13846 and *P. aeruginosa* PAO1 (ATCC 15692) was used as reference strain (Supplemental Material; Fig. 1).

### The mucoid phenotype

The mucoid phenotype was qualitatively assessed based on the mucoid appearance of the colony after 48 h of incubation in the MacConkey medium (bioMérieux, Marcy-l'Étoile France) at 37 °C.

### Pyocyanin

The production of pigment was evaluated using selective cetrimide agar (Merck, Germany) by visually assessing the color of cultured *P. aeruginosa* colonies^36^.

### Motility assays

Swimming, swarming and twitching were assessed using a 5xM8 solution supplemented with 20% glucose, 20% hydrolyzed casein and 1 M MgSO_4_. Swimming and swarming were measured on 0.3% swimming and 0.6% swarming agar plates, respectively. Twitching was assessed using 1% LB agar (Sigma-Aldrich, USA). Motility was evaluated after 24 h incubation at 37 °C by measuring the diameter of movement^37^.

### Alkaline protease

The proteolytic activity of the strains was determined using 10% skim milk powder (Sigma-Aldrich, USA) as a substrate. 5 µl of *P. aeruginosa* culture in the LB broth was applied to the medium and incubated for 24 h at 37 °C. The brightness surrounding the colony was assessed, and the zone diameter was measured^37, 38^.

### Elastase

The elastase activity was determined using a two-layer medium. The base was 0.8% nutrient broth (Becton Dickinson, USA) and 2.0% Noble Agar (Difco™ Agar Noble, Becton Dickinson, USA). The top layer contained 0.8% nutrient agar, 2.0% noble agar and 0.5% elastin (Elastin from bovine neck ligament, Sigma-Aldrich, USA). 5 µl of *P. aeruginosa* culture was applied to the medium and incubated for 48 h at 37 °C. The brightness surrounding the colony was assessed, and the zone diameter was measured^37, 39^.

### Microtitre plate assays for assessment of *P. aeruginosa* biofilm formation

Crystal violet (CV) staining and a validated MTT cell viability assay were used to assess biofilm to each *P. aeruginosa* strain. Unlike the widely used CV staining method, the MTT assay does not stain polysaccharides, proteins or DNA within the biofilm. Only live bacteria in the biofilms are counted in the MTT assay by measuring the metabolic activity of each individual bacterial cell. CV staining was used for the quantification of biofilm formation (biomass) while the MTT assay was utilized to evaluate the viability of bacteria in biofilms.

### Microtitre plate assays for assessment of *P. aeruginosa* biofilm formation using crystal violet

200 μl of the bacterial suspension was applied to sterile microtiter plates (FL MEDICAL). The plates were incubated at 22 °C for 24 h then washed nine times with PBS (Aqua-med). The resulting biofilm was fixed with 0.3% formalin solution and washed three times with PBS. In order to stain the biofilm, 200 µl of crystal violet solution (bioMérieux, France) was added. After 15 min, the pigment was removed by rinsing with dH_2_O, and 200 μl of 96% ethanol was added. Absorbance was measured spectrophotometrically at 595 nm (CLARIOstar^®^ Plus, BMG LABTECH)^40^.

### Microtitre plate assays for assessment of *P. aeruginosa* biofilm formation using 3-(4,5-di-methyl-2-thiazolyl)-2,5-diphenyl-2H tetrazolium bromide (MTT)

200 μl of the bacterial suspension was applied to sterile microtiter plates (FL MEDICAL). After incubation, bacterial planktonic cells were removed with a sterile pipette. 150 µl of PBS and 50 µl of 0.3% MTT (Sigma-Aldrich, USA) were added to the wells and incubated for 2 h at 37 °C. After this time, the MTT was removed, and 150 μl of dimethyl sulfoxide (DMSO, Sigma-Aldrich, USA) and 25 μl of glycine buffer were added. Absorbance was measured spectrophotometrically at 550 nm (CLARIOstar^®^ Plus, BMG LABTECH)^41^.

### Determination of drug resistance to antibiotics and chemotherapeutics

The tests of drug resistance to antibiotics and chemotherapeutics were performed by the qualitative diffusion method using paper discs saturated with an antibiotic (Becton Dickinson, USA). Colistin sensitivity was determined by microdilution in broth (MIC-Strip Colistin, MERLIN). Although the strains were isolated in the years 2012–2019, drug resistance results were interpreted in accordance with the current recommendations of the European Committee on Antimicrobial Susceptibility Testing in order to standardize the sensitivity categories of the strains^42^.

### Statistical analysis

Categorical variables were presented as the counts and percentages and compared between groups using the Pearson Chi-squared test or Fisher Exact test if the count in any cell of the contingency table was < 5. Continuous variables were presented as the median and range or individual measurements on diagrams, and compared between groups using the Mann–Whitney U test. A p-value < 0.05 was considered as indicative of statistical significance. Statistical analysis was performed in TIBCO Statistica 13.3 (TIBCO Software Inc., Palo Alto, CA, USA).

### Ethics committee approval

Consent of the Bioethics Committee KB74/2019 at the Institute of Tuberculosis and Lung Diseases regarding *Pseudomonas* strains isolated from cystic fibrosis patients includes.

- A statement confirming that all methods were carried out in accordance with relevant guidelines and regulations.
- A statement confirming that all experimental protocols have been approved by the designated institutional and/or licensing committee.
- Informed consent was obtained from all participants.

## Supplementary Information

Supplementary Figure 1.

Supplementary Table 1.

## Data Availability

Raw data were generated at the Institute of Department of Microbiology, National Tuberculosis and Lung Diseases Research Institute, Warsaw. Derived data supporting the findings of this study are available from the corresponding author on request.
