# Supplementary figures and images for: Phenotypic and genotypic characteristics of Pseudomonas aeruginosa isolated from cystic fibrosis patients with chronic infections
Source: Sci Rep. 2023 Jul 20;13:11741. doi: 10.1038/s41598-023-39005-9 (PMC10359326; doi:10.1038/s41598-023-39005-9)

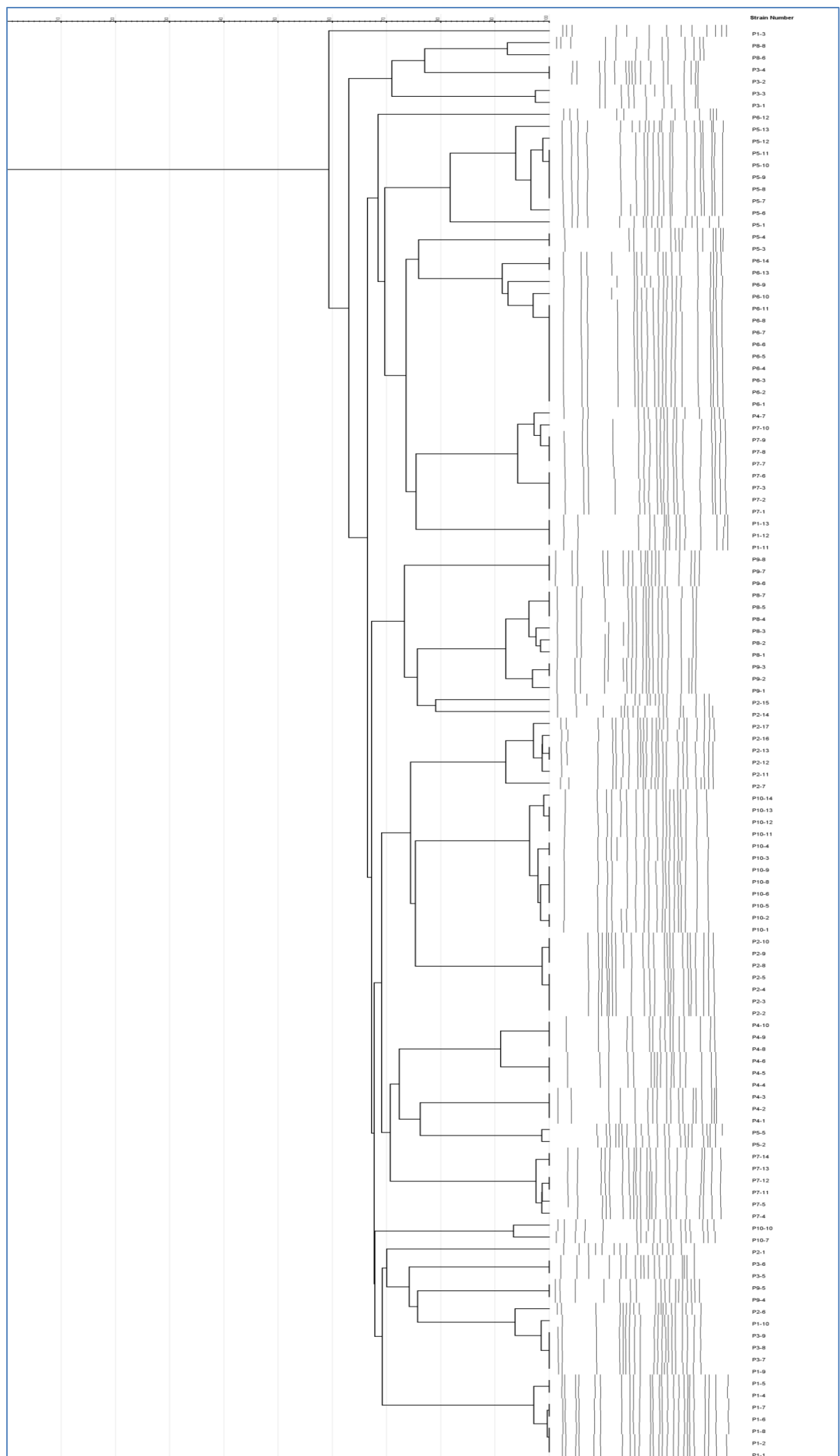

**Figure 1.** Dendrogram of 120 strains of *P. aeruginosa*.

Supplement: Supplementary file 1 — Supplementary Figure 1. [file 41598_2023_39005_MOESM1_ESM.pdf]
