# Supplementary material for: Phenotypic and genotypic characteristics of Pseudomonas aeruginosa isolated from cystic fibrosis patients with chronic infections
Source: Sci Rep. 2023 Jul 20;13:11741. doi: 10.1038/s41598-023-39005-9 (PMC10359326; doi:10.1038/s41598-023-39005-9)
Supplement: Supplementary file 2 — Supplementary Table 1. [file 41598_2023_39005_MOESM2_ESM.doc]

**Table 1**. Summary table of PFGE patterns and phenotypic characterisation of P. aeruginosa including mucoid/nonmucoid phenotype, biofilm CV, biofilm MTT, protease, elastase, swimming, swarming and twitching motility and pyocyanin production.

| **Number** | **Patient** | **Conventional strain number** | **PFGE pattern** | ***P. aeruginosa phenotype*** | **Biofilm CV - absorbance** | **Degree of CV biofilm formation** | **Biofilm MTT - absorbance** | **Degree of MTT biofilm formation** | **Protease** | | **Elastase** | | **Swimming**  **motility** | | **Swarming motility** | | **Twitching motility** | | **Pyocyanin** |
| --- | --- | --- | --- | --- | --- | --- | --- | --- | --- | --- | --- | --- | --- | --- | --- | --- | --- | --- | --- |
|  |  |  |  |  |  |  |  |  | **Diameter (mm)** | **Interpretation** | **Diameter (mm)** | **Interpretation** | **Diameter (mm)** | **Interprettation** | **Diameter (mm)** | **Interpretation** | **Diameter (mm)** | **Interpretation** | **Interpretation** |
| 1 | **P1** | P1-1 | AA | nonmucoid | 0,17 | none | 0,07 | none | 25 | positive | 20 | positive | 25 | positive | 5 | negative | 24 | positive | positive |
| 2 | **P1** | P1-2 | AA | nonmucoid | 0,5 | weak | 0,20 | none | 20 | positive | 22 | positive | 0 | negative | 4 | negative | 20 | positive | positive |
| 3 | **P1** | P1-3 | A | nonmucoid | 0,75 | moderate | 1,79 | strong | 15 | positive | 19 | positive | 0 | negative | 5 | negative | 15 | positive | negative |
| 4 | **P1** | P1-4 | AA | nonmucoid | 0,29 | none | 1,82 | strong | 14 | positive | 18 | positive | 26 | positive | 0 | negative | 15 | positive | positive |
| 5 | **P1** | P1-5 | AA | nonmucoid | 0,36 | none | 0,33 | none | 17 | positive | 16 | positive | 27 | positive | 0 | negative | 20 | positive | positive |
| 6 | **P1** | P1-6 | AA | nonmucoid | 0,45 | weak | 0,28 | none | 0 | negative | 17 | positive | 0 | negative | 0 | negative | 19 | positive | negative |
| 7 | **P1** | P1-7 | AA | nonmucoid | 0,26 | none | 0,21 | none | 0 | negative | 20 | positive | 0 | negative | 0 | negative | 16 | positive | positive |
| 8 | **P1** | P1-8 | AA | nonmucoid | 0,21 | none | 0,76 | moderate | 0 | negative | 21 | positive | 0 | negative | 0 | negative | 15 | positive | positive |
| 9 | **P1** | P1-9 | IG3 | nonmucoid | 0,19 | none | 0,38 | weak | 0 | negative | 0 | negative | 5 | negative | 0 | negative | 0 | negative | positive |
| 10 | **P1** | P1-10 | IG3 | nonmucoid | 0,21 | none | 0,33 | none | 0 | negative | 0 | negative | 0 | negative | 0 | negative | 3 | negative | negative |
| 11 | **P1** | P1-11 | Z | nonmucoid | 0,42 | weak | 0,25 | none | 14 | positive | 0 | negative | 37 | positive | 0 | negative | 17 | positive | negative |
| 12 | **P1** | P1-12 | Z | nonmucoid | 0,29 | none | 0,25 | none | 18 | positive | 0 | negative | 38 | positive | 0 | negative | 23 | positive | negative |
| 13 | **P1** | P1-13 | Z | nonmucoid | 0,83 | moderate | 1,87 | strong | 0 | negative | 22 | positive | 0 | negative | 0 | negative | 4 | negative | positive |
| 14 | **P2** | P2-1 | E | mucoid | 0,71 | weak | 1,56 | strong | 25 | positive | 0 | negative | 29 | positive | 22 | positive | 25 | positive | positive |
| 15 | **P2** | P2-2 | X | mucoid | 1,61 | strong | 2,44 | strong | 25 | positive | 20 | positive | 34 | positive | 20 | positive | 24 | positive | positive |
| 16 | **P2** | P2-3 | X | mucoid | 2,49 | strong | 2,84 | strong | 25 | positive | 21 | positive | 20 | positive | 25 | positive | 23 | positive | positive |
| 17 | **P2** | P2-4 | X | mucoid | 0,53 | weak | 1,97 | strong | 23 | positive | 20 | positive | 40 | positive | 15 | positive | 22 | positive | positive |
| 18 | **P2** | P2-5 | X | mucoid | 0,46 | weak | 1,02 | moderate | 23 | positive | 18 | positive | 40 | positive | 16 | positive | 15 | positive | negative |
| 19 | **P2** | P2-6 | IG3 | nonmucoid | 0,58 | weak | 1,95 | strong | 25 | positive | 22 | positive | 35 | positive | 19 | positive | 13 | positive | positive |
| 20 | **P2** | P2-7 | Y | mucoid | 0,23 | none | 1,08 | moderate | 23 | positive | 17 | positive | 17 | positive | 25 | positive | 22 | positive | negative |
| 21 | **P2** | P2-8 | X | nonmucoid | 0,58 | weak | 2,36 | strong | 25 | positive | 15 | positive | 35 | positive | 19 | positive | 3 | negative | positive |
| 22 | **P2** | P2-9 | X | nonmucoid | 0,25 | none | 0,36 | weak | 26 | positive | 19 | positive | 36 | positive | 25 | positive | 15 | positive | negative |
| 23 | **P2** | P2-10 | X | mucoid | 0,65 | weak | 2,10 | strong | 20 | positive | 16 | positive | 30 | positive | 25 | positive | 4 | negative | positive |
| 24 | **P2** | P2-11 | Y | nonmucoid | 0,48 | weak | 0,31 | none | 0 | negative | 17 | positive | 0 | negative | 0 | negative | 3 | negative | negative |
| 25 | **P2** | P2-12 | Y | mucoid | 0,23 | none | 0,14 | none | 23 | positive | 0 | negative | 25 | positive | 24 | positive | 2 | negative | negative |
| 26 | **P2** | P2-13 | Y | nonmucoid | 0,31 | none | 0,32 | none | 25 | positive | 15 | positive | 0 | negative | 19 | positive | 1 | negative | negative |
| 27 | **P2** | P2-14 | O | mucoid | 0,55 | weak | 0,88 | moderate | 15 | positive | 18 | positive | 5 | negative | 19 | positive | 4 | negative | positive |
| 28 | **P2** | P2-15 | N | nonmucoid | 0,13 | none | 0,12 | none | 0 | negative | 18 | positive | 0 | negative | 0 | negative | 4 | negative | positive |
| 29 | **P2** | P2-16 | Y | mucoid | 0,33 | none | 0,18 | none | 0 | negative | 19 | positive | 0 | negative | 0 | negative | 5 | negative | negative |
| 30 | **P2** | P2-17 | Y | nonmucoid | 0,21 | none | 0,09 | none | 0 | negative | 14 | positive | 0 | negative | 22 | positive | 0 | negative | positive |
| 31 | **P3** | P3-1 | D | mucoid | 0,33 | none | 1,24 | moderate | 20 | positive | 15 | positive | 42 | positive | 16 | positive | 17 | positive | negative |
| 32 | **P3** | P3-2 | C | mucoid | 0,35 | none | 2,00 | strong | 0 | negative | 18 | positive | 20 | positive | 0 | negative | 15 | positive | negative |
| 33 | **P3** | P3-3 | D | mucoid | 0,57 | weak | 0,37 | weak | 21 | positive | 22 | positive | 25 | positive | 22 | positive | 14 | positive | positive |
| 34 | **P3** | P3-4 | C | mucoid | 0,74 | moderate | 0,76 | moderate | 25 | positive | 17 | positive | 27 | positive | 24 | positive | 12 | positive | positive |
| 35 | **P3** | P3-5 | S | mucoid | 1,4 | moderate | 2,89 | strong | 0 | negative | 16 | positive | 46 | positive | 0 | negative | 20 | positive | positive |
| 36 | **P3** | P3-6 | S | mucoid | 0,3 | none | 0,39 | weak | 25 | positive | 20 | positive | 32 | positive | 17 | positive | 22 | positive | positive |
| 37 | **P3** | P3-7 | IG3 | mucoid | 0,2 | none | 0,38 | weak | 25 | positive | 19 | positive | 30 | positive | 17 | positive | 21 | positive | positive |
| 38 | **P3** | P3-8 | IG3 | mucoid | 0,18 | none | 0,29 | none | 22 | positive | 18 | positive | 25 | positive | 17 | positive | 16 | positive | positive |
| 39 | **P3** | P3-9 | IG3 | mucoid | 0,16 | none | 0,13 | none | 15 | positive | 16 | positive | 21 | positive | 17 | positive | 15 | positive | positive |
| 40 | **P4** | P4-1 | H | nonmucoid | 0,23 | none | 0,26 | none | 0 | negative | 0 | negative | 40 | positive | 16 | positive | 22 | positive | negative |
| 41 | **P4** | P4-2 | H | nonmucoid | 0,34 | none | 0,45 | weak | 0 | negative | 0 | negative | 30 | positive | 22 | positive | 19 | positive | negative |
| 42 | **P4** | P4-3 | H | nonmucoid | 0,88 | moderate | 3,48 | strong | 0 | negative | 0 | negative | 0 | negative | 22 | positive | 2 | negative | negative |
| 43 | **P4** | P4-4 | G | nonmucoid | 0,46 | weak | 0,16 | none | 0 | negative | 0 | negative | 30 | positive | 23 | positive | 13 | positive | negative |
| 44 | **P4** | P4-5 | G | nonmucoid | 0,26 | none | 0,16 | none | 0 | negative | 15 | positive | 5 | negative | 0 | negative | 4 | negative | negative |
| 45 | **P4** | P4-6 | G | nonmucoid | 0,89 | moderate | 3,20 | strong | 0 | negative | 0 | negative | 30 | positive | 21 | positive | 3 | negative | negative |
| 46 | **P4** | P4-7 | IG1 | nonmucoid | 0,77 | moderate | 2,42 | strong | 0 | negative | 19 | positive | 30 | positive | 18 | positive | 0 | negative | positive |
| 47 | **P4** | P4-8 | G | nonmucoid | 2,25 | strong | 2,77 | strong | 0 | negative | 0 | negative | 0 | negative | 0 | negative | 3 | negative | negative |
| 48 | **P4** | P4-9 | G | nonmucoid | 0,24 | none | 0,13 | none | 0 | negative | 0 | negative | 5 | negative | 0 | negative | 5 | negative | negative |
| 49 | **P4** | P4-10 | G | nonmucoid | 0,82 | moderate | 1,31 | moderate | 0 | negative | 16 | positive | 25 | positive | 16 | positive | 15 | positive | negative |
| 50 | **P5** | P5-1 | J | mucoid | 0,3 | none | 1,06 | moderate | 23 | positive | 14 | positive | 23 | positive | 16 | positive | 3 | negative | positive |
| 51 | **P5** | P5-2 | U | mucoid | 0,88 | moderate | 2,15 | strong | 25 | positive | 19 | positive | 28 | positive | 25 | positive | 5 | negative | positive |
| 52 | **P5** | P5-3 | K | mucoid | 0,2 | none | 0,11 | none | 22 | positive | 0 | negative | 31 | positive | 16 | positive | 3 | negative | negative |
| 53 | **P5** | P5-4 | K | nonmucoid | 0,4 | weak | 0,23 | none | 25 | positive | 0 | negative | 30 | positive | 19 | positive | 4 | negative | negative |
| 54 | **P5** | P5-5 | U | mucoid | 1,34 | moderate | 1,24 | moderate | 0 | negative | 0 | negative | 2 | negative | 0 | negative | 0 | negative | negative |
| 55 | **P5** | P5-6 | I | mucoid | 0,47 | weak | 1,88 | strong | 25 | positive | 0 | negative | 33 | positive | 20 | positive | 4 | negative | negative |
| 56 | **P5** | P5-7 | I | nonmucoid | 0,69 | weak | 0,82 | moderate | 20 | positive | 0 | negative | 33 | positive | 25 | positive | 4 | negative | negative |
| 57 | **P5** | P5-8 | I | mucoid | 0,15 | none | 0,30 | none | 23 | positive | 0 | negative | 35 | positive | 18 | positive | 3 | negative | negative |
| 58 | **P5** | P5-9 | I | mucoid | 1,34 | moderate | 0,91 | moderate | 20 | positive | 0 | negative | 31 | positive | 18 | positive | 4 | negative | negative |
| 59 | **P5** | P5-10 | I | mucoid | 1,77 | strong | 1,60 | strong | 0 | negative | 0 | negative | 5 | negative | 25 | positive | 0 | negative | negative |
| 60 | **P5** | P5-11 | I | mucoid | 0,19 | none | 0,31 | none | 0 | negative | 0 | negative | 0 | negative | 22 | positive | 4 | negative | negative |
| 61 | **P5** | P5-12 | I | nonmucoid | 1,23 | moderate | 0,60 | weak | 0 | negative | 0 | negative | 0 | negative | 0 | negative | 4 | negative | negative |
| 62 | **P5** | P5-13 | I | mucoid | 0,25 | none | 0,15 | none | 0 | negative | 0 | negative | 15 | positive | 19 | positive | 3 | negative | negative |
| 63 | **P6** | P6-1 | L | mucoid | 0,42 | weak | 2,31 | strong | 14 | positive | 15 | positive | 28 | positive | 0 | negative | 20 | positive | positive |
| 64 | **P6** | P6-2 | L | mucoid | 2,1 | strong | 3,09 | strong | 25 | positive | 15 | positive | 20 | positive | 0 | negative | 20 | positive | positive |
| 65 | **P6** | P6-3 | L | nonmucoid | 1,36 | moderate | 3,35 | strong | 15 | positive | 15 | positive | 20 | positive | 4 | negative | 20 | positive | positive |
| 66 | **P6** | P6-4 | L | nonmucoid | 1,23 | moderate | 2,25 | strong | 16 | positive | 16 | positive | 3 | negative | 3 | negative | 19 | positive | positive |
| 67 | **P6** | P6-5 | L | nonmucoid | 0,97 | moderate | 2,52 | strong | 15 | positive | 20 | positive | 5 | negative | 5 | negative | 17 | positive | positive |
| 68 | **P6** | P6-6 | L | nonmucoid | 0,18 | none | 0,16 | none | 16 | positive | 21 | positive | 0 | negative | 0 | negative | 19 | positive | negative |
| 69 | **P6** | P6-7 | L | nonmucoid | 0,17 | none | 0,15 | none | 25 | positive | 19 | positive | 5 | negative | 0 | negative | 15 | positive | negative |
| 70 | **P6** | P6-8 | L | nonmucoid | 0,2 | none | 0,22 | none | 25 | positive | 16 | positive | 3 | negative | 0 | negative | 22 | positive | negative |
| 71 | **P6** | P6-9 | L | nonmucoid | 0,17 | none | 0,38 | weak | 0 | negative | 17 | positive | 5 | negative | 0 | negative | 15 | positive | negative |
| 72 | **P6** | P6-10 | L | nonmucoid | 1,64 | strong | 3,2 | strong | 14 | positive | 15 | positive | 2 | negative | 0 | negative | 21 | positive | positive |
| 73 | **P6** | P6-11 | L | nonmucoid | 1,39 | moderate | 3,4 | strong | 17 | positive | 19 | positive | 5 | negative | 5 | negative | 25 | positive | positive |
| 74 | **P6** | P6-12 | F | nonmucoid | 0,78 | moderate | 2,79 | strong | 0 | negative | 16 | positive | 25 | positive | 0 | negative | 0 | negative | negative |
| 75 | **P6** | P6-13 | L | nonmucoid | 0,87 | moderate | 0,8 | moderate | 16 | positive | 16 | positive | 35 | positive | 3 | negative | 23 | positive | positive |
| 76 | **P6** | P6-14 | L | mucoid | 2,17 | strong | 0,8 | moderate | 0 | negative | 22 | positive | 0 | negative | 4 | negative | 20 | positive | positive |
| 77 | **P7** | P7-1 | IG1 | mucoid | 1,61 | strong | 2,68 | strong | 0 | negative | 17 | positive | 29 | positive | 16 | positive | 17 | positive | positive |
| 78 | **P7** | P7-2 | IG1 | mucoid | 1,6 | strong | 2,87 | strong | 0 | negative | 16 | positive | 20 | positive | 22 | positive | 16 | positive | positive |
| 79 | **P7** | P7-3 | IG1 | mucoid | 1,03 | moderate | 2,48 | strong | 17 | positive | 18 | positive | 20 | positive | 20 | positive | 20 | positive | positive |
| 80 | **P7** | P7-4 | P | nonmucoid | 0,3 | none | 0,29 | none | 0 | negative | 0 | negative | 30 | positive | 25 | positive | 24 | positive | positive |
| 81 | **P7** | P7-5 | P | mucoid | 0,37 | weak | 0,33 | none | 16 | positive | 0 | negative | 25 | positive | 20 | positive | 24 | positive | positive |
| 82 | **P7** | P7-6 | IG1 | mucoid | 1,42 | moderate | 3,03 | strong | 14 | positive | 17 | positive | 22 | positive | 24 | positive | 20 | positive | positive |
| 83 | **P7** | P7-7 | IG1 | nonmucoid | 0,36 | none | 0,38 | weak | 20 | positive | 16 | positive | 15 | positive | 21 | positive | 20 | positive | positive |
| 84 | **P7** | P7-8 | IG1 | nonmucoid | 0,8 | moderate | 1,98 | strong | 0 | negative | 16 | positive | 40 | positive | 20 | positive | 20 | positive | positive |
| 85 | **P7** | P7-9 | IG1 | nonmucoid | 0,88 | moderate | 0,42 | weak | 18 | positive | 19 | positive | 28 | positive | 16 | positive | 17 | positive | positive |
| 86 | **P7** | P7-10 | IG1 | nonmucoid | 0,89 | moderate | 0,41 | weak | 16 | positive | 15 | positive | 30 | positive | 23 | positive | 15 | positive | positive |
| 87 | **P7** | P7-11 | P | nonmucoid | 0,78 | moderate | 0,53 | weak | 0 | negative | 0 | negative | 4 | negative | 16 | positive | 1 | negative | negative |
| 88 | **P7** | P7-12 | P | mucoid | 0,32 | none | 1,29 | moderate | 0 | negative | 0 | negative | 5 | negative | 2 | negative | 1 | negative | positive |
| 89 | **P7** | P7-13 | P | nonmucoid | 0,13 | none | 0,18 | none | 16 | positive | 18 | positive | 5 | negative | 0 | negative | 3 | negative | positive |
| 90 | **P7** | P7-14 | P | mucoid | 0,14 | none | 0,29 | none | 16 | positive | 18 | positive | 0 | negative | 5 | negative | 5 | negative | positive |
| 91 | **P8** | P8-1 | IG2 | nonmucoid | 1,52 | strong | 2,96 | strong | 14 | positive | 22 | positive | 0 | negative | 4 | negative | 16 | positive | positive |
| 92 | **P8** | P8-2 | IG2 | nonmucoid | 1,27 | moderate | 3,33 | strong | 15 | positive | 0 | negative | 3 | negative | 2 | negative | 15 | positive | negative |
| 93 | **P8** | P8-3 | IG2 | nonmucoid | 0,17 | none | 0,32 | none | 20 | positive | 0 | negative | 2 | negative | 5 | negative | 16 | positive | negative |
| 94 | **P8** | P8-4 | IG2 | nonmucoid | 0,28 | none | 0,44 | weak | 15 | positive | 17 | positive | 2 | negative | 0 | negative | 17 | positive | positive |
| 95 | **P8** | P8-5 | IG2 | nonmucoid | 0,33 | none | 0,64 | weak | 0 | negative | 15 | positive | 2 | negative | 0 | negative | 23 | positive | negative |
| 96 | **P8** | P8-6 | B | nonmucoid | 0,14 | none | 0,16 | none | 0 | negative | 18 | positive | 0 | negative | 0 | negative | 14 | positive | negative |
| 97 | **P8** | P8-7 | IG2 | nonmucoid | 0,17 | none | 0,18 | none | 0 | negative | 16 | positive | 0 | negative | 0 | negative | 14 | positive | negative |
| 98 | **P8** | P8-8 | B | nonmucoid | 0,24 | none | 0,32 | none | 0 | negative | 16 | positive | 3 | negative | 0 | negative | 15 | positive | negative |
| 99 | **P9** | P9-1 | IG2 | mucoid | 0,99 | moderate | 1,80 | strong | 15 | positive | 16 | positive | 2 | negative | 16 | positive | 14 | positive | positive |
| 100 | **P9** | P9-2 | IG2 | mucoid | 0,92 | moderate | 2,92 | strong | 15 | positive | 19 | positive | 15 | positive | 19 | positive | 20 | positive | positive |
| 101 | **P9** | P9-3 | IG2 | nonmucoid | 1,17 | moderate | 1,77 | strong | 18 | positive | 17 | positive | 15 | positive | 20 | positive | 23 | positive | positive |
| 102 | **P9** | P9-4 | T | nonmucoid | 0,81 | moderate | 1,19 | moderate | 14 | positive | 15 | positive | 20 | positive | 19 | positive | 5 | negative | positive |
| 103 | **P9** | P9-5 | T | nonmucoid | 0,8 | moderate | 1,27 | moderate | 16 | positive | 16 | positive | 19 | positive | 16 | positive | 3 | negative | positive |
| 104 | **P9** | P9-6 | M | nonmucoid | 0,42 | weak | 0,44 | weak | 0 | negative | 19 | positive | 17 | positive | 16 | positive | 5 | negative | positive |
| 105 | **P9** | P9-7 | M | nonmucoid | 0,8 | moderate | 1,61 | strong | 0 | negative | 18 | positive | 16 | positive | 18 | positive | 0 | negative | positive |
| 106 | **P9** | P9-8 | M | nonmucoid | 0,2 | none | 1,21 | moderate | 0 | negative | 15 | positive | 22 | positive | 23 | positive | 4 | negative | positive |
| 107 | **P10** | P10-1 | W | nonmucoid | 0,3 | none | 0,32 | none | 0 | negative | 15 | positive | 25 | positive | 23 | positive | 20 | positive | positive |
| 108 | **P10** | P10-2 | W | nonmucoid | 0,49 | weak | 0,33 | none | 0 | negative | 21 | positive | 30 | positive | 18 | positive | 20 | positive | positive |
| 109 | **P10** | P10-3 | W | nonmucoid | 0,5 | weak | 0,31 | none | 0 | negative | 17 | positive | 35 | positive | 20 | positive | 21 | positive | negative |
| 110 | **P10** | P10-4 | W | mucoid | 1,69 | strong | 3,50 | strong | 0 | negative | 19 | positive | 29 | positive | 21 | positive | 25 | positive | positive |
| 111 | **P10** | P10-5 | W | nonmucoid | 0,48 | weak | 0,38 | weak | 0 | negative | 15 | positive | 40 | positive | 19 | positive | 18 | positive | positive |
| 112 | **P10** | P10-6 | W | mucoid | 1,04 | moderate | 3,04 | strong | 0 | negative | 18 | positive | 30 | positive | 20 | positive | 20 | positive | negative |
| 113 | **P10** | P10-7 | R | nonmucoid | 0,16 | none | 0,31 | none | 0 | negative | 16 | positive | 40 | positive | 20 | positive | 5 | negative | negative |
| 114 | **P10** | P10-8 | W | nonmucoid | 0,92 | moderate | 2,45 | strong | 0 | negative | 17 | positive | 40 | positive | 23 | positive | 4 | negative | negative |
| 115 | **P10** | P10-9 | W | nonmucoid | 1,18 | moderate | 2,24 | strong | 0 | negative | 15 | positive | 45 | positive | 18 | positive | 3 | negative | negative |
| 116 | **P10** | P10-10 | R | mucoid | 0,49 | weak | 0,18 | none | 0 | negative | 19 | positive | 20 | positive | 18 | positive | 3 | negative | negative |
| 117 | **P10** | P10-11 | W | nonmucoid | 0,23 | none | 0,96 | moderate | 0 | negative | 17 | positive | 20 | positive | 20 | positive | 20 | positive | positive |
| 118 | **P10** | P10-12 | W | nonmucoid | 0,5 | weak | 0,85 | moderate | 0 | negative | 16 | positive | 20 | positive | 23 | positive | 20 | positive | positive |
| 119 | **P10** | P10-13 | W | nonmucoid | 0,32 | none | 0,37 | weak | 0 | negative | 19 | positive | 25 | positive | 18 | positive | 4 | negative | positive |
| 120 | **P10** | P10-14 | W | mucoid | 0,24 | none | 0,25 | none | 0 | negative | 22 | positive | 20 | positive | 23 | positive | 4 | negative | positive |
